# MULTI-K: accurate classification of microarray subtypes using ensemble k-means clustering

**DOI:** 10.1186/1471-2105-10-260

**Published:** 2009-08-22

**Authors:** Eun-Youn Kim, Seon-Young Kim, Daniel Ashlock, Dougu Nam

**Affiliations:** 1 National Institute for Mathematical Sciences (NIMS), Yuseong, Daejeon 305–340, Republic of Korea; 2 Korea Research Institute of Bioscience and Biotechnology (KRIBB), PO Box 115, Yuseong, Daejeon 305–600, Republic of Korea; 3 Department of Mathematics and Statistics, University of Guelph, Ontario, N1G 2R4, Canada

## Abstract

**Background:**

Uncovering subtypes of disease from microarray samples has important clinical implications such as survival time and sensitivity of individual patients to specific therapies. Unsupervised clustering methods have been used to classify this type of data. However, most existing methods focus on clusters with compact shapes and do not reflect the geometric complexity of the high dimensional microarray clusters, which limits their performance.

**Results:**

We present a cluster-number-based ensemble clustering algorithm, called *MULTI-K*, for microarray sample classification, which demonstrates remarkable accuracy. The method amalgamates multiple *k*-means runs by varying the number of clusters and identifies clusters that manifest the most robust co-memberships of elements. In addition to the original algorithm, we newly devised the *entropy-plot *to control the separation of singletons or small clusters. MULTI-K, unlike the simple *k*-means or other widely used methods, was able to capture clusters with complex and high-dimensional structures accurately. MULTI-K outperformed other methods including a recently developed ensemble clustering algorithm in tests with five simulated and eight real gene-expression data sets.

**Conclusion:**

The geometric complexity of clusters should be taken into account for accurate classification of microarray data, and ensemble clustering applied to the number of clusters tackles the problem very well. The C++ code and the data sets tested are available from the authors.

## Background

Groups that exhibit similar patterns in large-scale genomic data sets have provided primary biological information. In this regard, identification of natural clusters and their membership has excited a great deal of interest in functional genomics and clinical research. Indeed, unsupervised clustering methods applied to microarray data enabled predictions of unknown gene functions (if applied to genes) [1,2] and suggested the existence of subtypes of disease (if applied to samples) [3-6]. The task of cluster identification heavily depends on the property of clusters that are of interest, e.g., compactness, connectedness, and spatial separation. Each clustering algorithm has pros and cons for different shapes of clusters, which in turn informs the choice of an appropriate clustering strategy [7].

We are interested in establishing subclasses among microarray samples that might enable specified clinical treatments. In this problem, the data points are distributed in a very high dimensional (hundreds or thousands) space and the geometry of their clusters is mostly uncharacterized, which make it difficult to choose an appropriate clustering method. However, the most widely used clustering methods for this problem have been the hierarchical agglomerative or *k*-means clustering algorithms [4,5,8,9] that mainly focus on clusters with compact shapes.

When using these methods, various internal measures that represent compactness and spatial separation of clusters have been developed and compared to identify clusters and their members in data sets [9-12]. Each of the measures, however, is prone to specific biases [7], and their tests were mainly focused on the ability to identify the number of clusters and not on the accuracy of classification, which is mainly attributed to the clustering strategies used.

One important line of effort to improve the clustering strategy is the development of ensemble or consensus clustering techniques. These methods amalgamate multiple clustering runs to capture robust cluster structures by using multiple clustering algorithms [13,14], perturbing data values with noise [15,16], using different gene subsets [17-19], or choosing the number of clusters randomly [20,21] and then extract consensus cluster structures. Among them, two of the methods [16,19] have been tested intensively on microarray sample classification and were compared favorably with previous methods.

In this article, we firstly apply a cluster-number-based ensemble technique for microarray sample classification and compare the performance with previously used methods. The advantage of this approach over the single clustering or other methods is the ability to capture complex geometric structures. The rationale is that since some large cluster numbers are chosen during the clustering process, co-memberships among detailed local structures are strengthened. See also [22] for developments of related algorithms. Specifically, we use the multiple *k*-means clustering by Ashlock *et al*. [20], dubbed *MULTI-K*, that provides most simple and intuitive procedure for partitioning data. In addition to the original algorithm, we newly devised an entropy-based analysis of clusters, called the *entropy-plot*, that monitors the distribution of cluster sizes during the partition process. The entropy-plot helps prevent singletons or very small clusters from forming separate clusters, of particular utility when analyzing high-dimensional and noisy real expression data.

MULTI-K, though it is the simplest among existing ensemble clustering methods, exhibited remarkable performance, surpassing previously used methods in our tests, which suggests its ability to classify complex geometric structures is an important factor for microarray sample classification. In particular, MULTI-K demonstrated perfect classification for five (or six) gene expression data sets out of eight that we tested.

## Results

### Algorithm

We begin by describing the MULTI-K algorithm. MULTI-K is performed by applying the well-known *k*-means algorithm repeatedly. Euclidean distance is always used to measure the dissimilarity between two data points unless stated otherwise. Let *S *= {*x*_1_, *x*_2_,..., *x*_*N*_} be the data set distributed in **R**^*n*^. The algorithm constructs an edge-weighted graph from the output of multiple instances of the *k*-means algorithm. MULTI-K is largely composed of the following two steps.

#### Step 1

Apply the *k*-means algorithm on the data *M *times. In each instance, we randomly sample a number *k*_*i*_, for the number of clusters, from a pre-determined distribution *D*. On each pair of nodes (edge) that belong to the same cluster, we add one to an edge weight (these weights are initialized to zero). After we repeat the process *M *times, we obtain a weighted graph structure on the data.

#### Step 2

Now, we go back to the reverse direction by unweighting the graph *M *times. In each iteration, we reduce a unit weight for all the edges with positive weights simultaneously. Through this reverse process, the initial graph is divided into smaller and smaller sub-graphs (clusters). At any point the connected components of the graph are the clusters. The plot between the discrete (reverse) time normalized by *M *and the number of divided sub-graphs, as we call *cut-plot*, provides the information on the natural number of clusters. If a flat region of the cut-plot is long, we regard the corresponding cluster structure is robust and hence natural. We choose the longest interval in the cut-plot for the predicted number of clusters except for the one-cluster interval. The weighted graph in Step 1 is equivalent to the averaged co-membership matrix used in other ensemble clustering algorithms [16,19]. The convergence of MULTI-K (in probability) is addressed in Methods.

### Entropy-plot

Entropy can be used as a measure of randomness or information in a set of clusters [23] and is defined in terms of a discrete random event *x*, with possible states *1*,..., *k *as: *H*(*x*) = -Σ*P*_*i*_log_2_*P*_*i*_, where *P*_*i *_= **Prob **(*x *= *i*th state), *i *= 1, 2,..., *k*. Let *S *be a data set with *N *elements and *X *= {*C*_1_, *C*_2_,... *C*_*k*_} be a set of non-overlapping clusters. The empirical probability that *C*_*i *_includes a given data point is |*C*_*i*_|/*N*, and so the entropy of the clustering *X *is



This entropy measure informs us how the data points are distributed as clusters. The cut-plot summarizes the hierarchical structure of clusters that form as the cut-value (where-to-cut in the cut-plot) is changed. However, the cut-plot alone does not distinguish between roughly-equal and substantially unequal divisions of clusters within the hierarchy. The entropy-plot is a more informative plot that better summarizes the cluster structure. This plot displays the Shannon-entropy of the empirical distribution of points into clusters as a function of the cut-value. In each position where the cut-plot jumps, the entropy-plot jumps as well. The difference lies in the size of the jumps. In the cut-plot, any division of a cluster yields a jump of height one; the entropy-plot has variable height jumps which give the relative informativeness of the partitioning of clusters. An even division of a large cluster is highly informative while the separation of a single point is minimally informative. When working with clean and low-dimensional data, it is not too difficult to detect the separation of small clusters consisting of one or a few points by inspection. When dealing with noisy or high-dimensional data such as gene expression profiles, the entropy-plot is of substantial utility in highlighting the significant divisions within the cluster structure. As a convenience for the user, the partition of small clusters, those for which the increase in entropy is less than a threshold, may be suppressed. This yields a cleaner and more easily interpreted summary of the hierarchical cluster structure. Users that wish to see the unmodified cluster structure may reduce the threshold value that triggers suppression. However, we applied a fixed threshold in this paper to compare the performance of MULTI-K with other algorithms. See **Additional file **1 for further explanation of entropy-plot.

### MULTI-K parameters

Since MULTI-K is an ensemble learning algorithm, it requires some parameters or thresholds. The following initial setting is suggested from our rough estimation and empirical tests, but works well for analyzing real-world expression data. Although we suggest varying them around the given values in an explorative study, we used the following setting for the purpose of comparison throughout this study.

The distribution for the cluster numbers, *D *is simply chosen to be uniform on an interval between two integers in our study. We used the interval (min(5, [*N*/4]), min(20, [*N*/2])) for *D*, where *N *is the number of samples and [x] represents rounding x to the nearest integer. We applied the minimum function here because it may be unreasonable to expect five or more classes for very small number of samples. The number of clustering runs *M *is fixed at 200 in this study. This number is sufficiently large for convergence of the algorithm. Lastly, we used a threshold for the entropy-plot such that if a jump in the entropy-plot is smaller than 0.1/(increased number of clusters), we suppress the separation of the corresponding cluster and merge the two adjacent intervals in the cut-plot. 'increased number of clusters' in the denominator accounts for the case when multiple singleton or small sets are separated simultaneously from a cluster.

### Experiments

We compare the performance of four kinds of clustering algorithms: MULTI-K, hierarchical clustering (average linkage), *k*-means, and *GCC*, a gene-subset based ensemble clustering [19] for classifying data points on various simulated and real expression data sets. We used code from the R package for hierarchical and *k*-means methods as well as for computing Silhuette Width and gap statistic. We tested the two versions GCC algorithms and denote them *GCCc *and *GCCk *that employ correlation and *k*-means clustering, respectively. These are all distance-based clustering methods. Another important class of algorithms that we did not consider is model-based clustering [24-26]. Most model-based methods, however, are designed mainly for gene clustering and may not be reliable for sample clustering because in most cases, the number of samples is not sufficiently high to fit very high-dimensional models. For example, EMMIX-GENE [25] reduces the number of genes when clustering samples, which suffers from significant information loss, and the class prediction is highly affected by the genes chosen.

We predict the number of classes in a data set as well as the cluster-membership of the data points in each algorithm, and then assess the agreement between the predicted and the known partitions using the adjusted Rand index (ARI) [27,28]. Let *P*_*I *_= {*P*_1•_, *P*_2•_,..., *P*_*l*•_} and *P*_*J *_= {*P*_•1_, *P*_•2_,..., *P*_•*m*_} be two partitions of a data set *D*. Let *N*_*i*• _and *N*_•*j *_be the number of elements in *P*_*i*• _and *P*_•*j*_, respectively and [*N*_*ij*_], be a *l *× *m *matrix where *N*_*ij *_represents the number of elements in *P*_*i*• _∩ *P*_•*j*_, then the ARI is computed as follows:



where *N *is the number of data points, i.e. the sum of [*N*_*ij*_]. This index addresses the degree of agreement between two partitions with possibly different numbers of clusters. ARI has a value between 0 and 1, which mean a random matching and the perfect agreement between the two partitions, respectively. The index is said to be 'adjusted' because its formulation compensates for the fact that, when there are more than two members of a partition, a majority of the pairs of data items are in distinct classes. We applied the gap statistic and Silhouette Width to hierarchical and *k*-means algorithms to predict the number of clusters, and used the inherent indicators for MULTI-K and GCC, the cut & entropy plot and the modified Rand index, respectively.

Most existing clustering algorithms are good at finding compact clusters, but not those interwoven among others. Combining multiple *k*-means runs, MULTI-K aims to find connected components in a data set that are spatially separated among others. Intuitive examples that characterize MULTI-K follow.

### Comparison for geometrically complicated clusters

**Figure 1:**
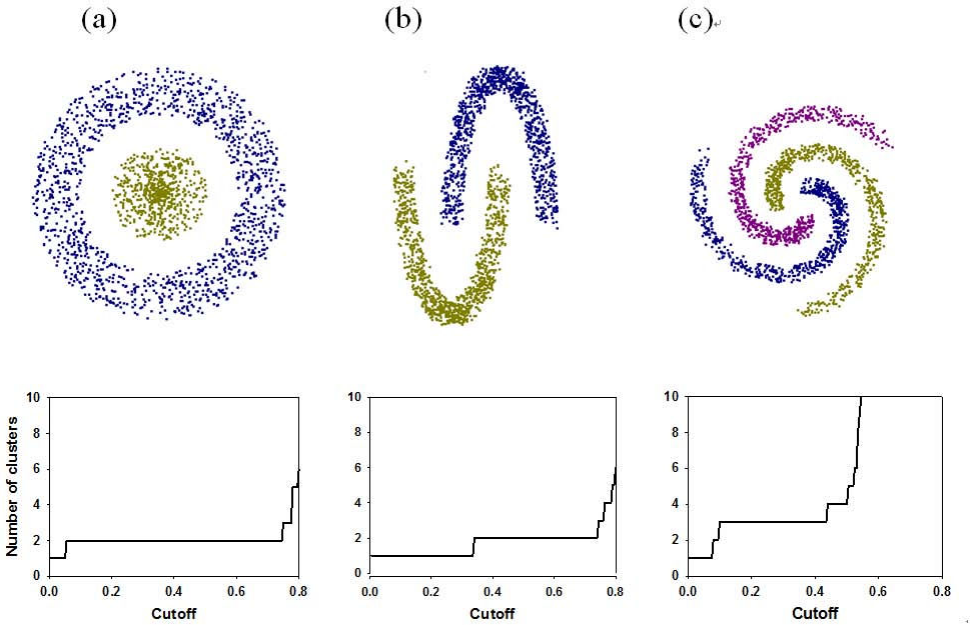
**The data sets with complicated clusters**: (a) Donut & Ball, (b) Horse Shoe, and (c) Spiral and their cut-plots.

We considered three data sets composed of clusters with geometrically complicated structures and named them Donut & Ball, Horse Shoe, and Spiral, respectively. Their shapes and the corresponding cut-plots are shown in Figure 1. All the three data sets have 1,000 data points. Although these data sets seem to have little relevance with gene expression data, they may abstract the geometric complexity of microarray data sets and clearly reveal the advantage of MULTI-K algorithm. The test results are shown in Table 1. The optimal number of clusters by MULTI-K is determined at the longest interval of the cut-plot (except *k *= 1 case), and then the corresponding partitions are naturally derived from Step 2. MULTI-K correctly predicted the number of clusters and classified the clusters accurately with ARI value 1 in all the three examples, while the other methods yielded poor predictions. Since most existing clustering algorithms and indicators for the optimal number of clusters are focused on compactness of clusters, they could not identify complicated structures that focused on connectedness and spatial separation of clusters as the above examples.

**Table 1:** The ARI values for the geometric data sets.

**Data set**		**Donut & Ball (2)**	**Horse-Shoe (2)**	**Spiral (3)**
**MULTI-K** **Cut & Entropy)**		**1.0000 (2)**	**1.0000 (2)**	**1.0000 (3)**

**GCCc**		0.6018 (2)	0 (2)	0.0510 (2)

**GCCk**		0.2553 (6)	0.1219 (5)	0.1487 (6)

**Hier**.	**gap**	0.2893 (10)	0.2596 (11)	0.2303 (20)
	
	**Silhuette**	0.4895 (2)	0.3434 (6)	0.1532 (29)

***k*-means**	**gap**	0.1752 (12)	0.3080 (7)	0.2072 (18)
	
	**Silhuette**	**0.7019 **(**2**)	0.3326 (6)	0.1460 (29)

In each parenthesis is shown the known (top header) and the predicted number of clusters. Those ARI values that exceed 0.7 are shown in bold.

The ability of MULTI-K to trace the complex geometric structure is reminiscent of the nonlinear dimension reduction technique, called ISOMAP [29], which uses the shortest paths between data points to approximate the geodesic distances between points, and may suggest the applicability of MULTI-K in nonlinear dimension reduction problem.

### Comparison for high dimensional and noisy clusters

Now, we compare the clustering algorithms on high dimensional and noisy synthetic data sets that imitate microarray samples. In the first model, called H2, we considered two clusters in 100 dimensions. Each cluster is chosen to have 25 or 50 observations that were independently drawn from normal distributions *N*(0_100_, *I*_100_) and *N*(0.5_100_, *I*_100_), respectively, where *α*_*p *_denotes the 1 by *p *vector of *α*'s and *I*_*p *_denotes the *p *by *p *identity (covariance) matrix. The two clusters may overlap in each dimension. In the second model, called H3, we considered three clusters in 300 dimensions (genes). Each cluster had 50 observations. We divided the 300 genes into ten groups each of which equally had 30 genes. In each block (a gene group in a cluster), all the 30 dimensional samples were commonly drawn from a normal distribution *N*(*α*_30_, *I*_30_) independently where *α *is randomly chosen from {-0.5, 0, 0.5} in each block. The block structures represent gene sets with co-expression patterns that are commonly up or down regulated under specific experimental conditions.

In each model, we tested the algorithms on randomly generated ten data sets and averaged the ARI values. The test results are summarized in Table 2. In both models, MULTI-K showed the highest accuracy. The GCC methods yielded rather good predictions for the H3 model, but performed very poorly for the H2 model. Hierarchical methods performed very poorly for all the cases, which is mostly attributed to the failure in predicting the correct number of clusters. *k*-means algorithm demonstrated the second best accuracy except for the case of the Silhuette indicator applied to the H3 model.

**Table 2:** Test results for high dimensional synthetic data sets.

**Data set**		**H2**	**H3**
**MULTI-K (Cut & Entropy)**		**0.9210 **(9/10)	**0.8695 **(7/10)

**GCCk**		0.5367 (5/10)	**0.7456 **(8/10)

**GCCc**		0.0993 (0/10)	**0.7799 **(10/10)

**Hier**.	**gap**	0 (1/10)	0.2277 (1/10)

	**Silhuette**	0.0839 (9/10)	0.3444 (4/10)

***k*-means**	**gap**	**0.7138 **(5/10)	**0.8433 **(7/10)
	
	**Silhuette**	**0.8445 **(9/10)	0.3715 (1/10)

The adjusted Rand indexes are shown averaged over ten randomly generated data sets. In each parenthesis is shown the number of cases out of ten that correctly identified the number of clusters. ARI values that exceed 0.7 are shown in bold.

### Classification tests for real expression data sets

We tested the clustering algorithms on eight microarray data sets as summarized in Table 3. All the mRNA samples in each data set are assigned class labels from laboratory analyses of the tumor samples. These labels establish the known (gold-standard) partitions on the data points. We chose 300 genes with higher variances in each data set for data clustering (or partitioning). For randomized algorithms, MULTI-K, GCC and *k*-means clustering, we repeated running the algorithms five times and used the median ARI and the corresponding clusters.

**Table 3:** Description of microarray data sets tested

Data set	Acquisition	Description of known subclasses	DNA Chip
Leukemia		ALL B-cell (38), ALL T-cell (9), AML (25)	Affymetrix HuFL
Lymphoma	GEO (GSE60)	B-CLL (12), FL (9), DLBCL (68)	cDNA
Colon tumor	GEO (GSE5206)	Colon tumor (100), Normal colon (5)	Affymetrix U133 Plus 2.0
Thyroid tumor I	GEO (GSE3467)	Thyroid tumor (9), Normal thyroid (9)	Affymetrix U133 Plus 2.0
Thyroid tumor II	GEO (GSE3678)	Thyroid tumor (7), Normal thyroid (7)	Affymetrix U133 Plus 2.0
St. Jude		BCR-ABL (15), E2A-PBX1 (27), Hyperdip-50 (64), MLL (20), T-ALL (43), TEL-AML1 (79)	Affymetrix U95A
Normal tissue I	GEO (GDS422)	Bone (2), Liver (2), Heart (2), Spleen (2), Lung (2), Kidney (2), Skeletal (2), Thymus (2), Brain (2), Spinal (2), Prostate (2), Pancreas (2)	Affymetrix U95A
Normal tissue II		Bladder (7), Breast (5), Cerebellum (3), Colon (11), Germinal Center (6), Kidney (12), Lung (7), Ovary (4), Pancreas (10), PBM (5), Prostate (9), Uterus (6), Whole Brain (5)	Affymetrix HuFL

**Figure 2:**
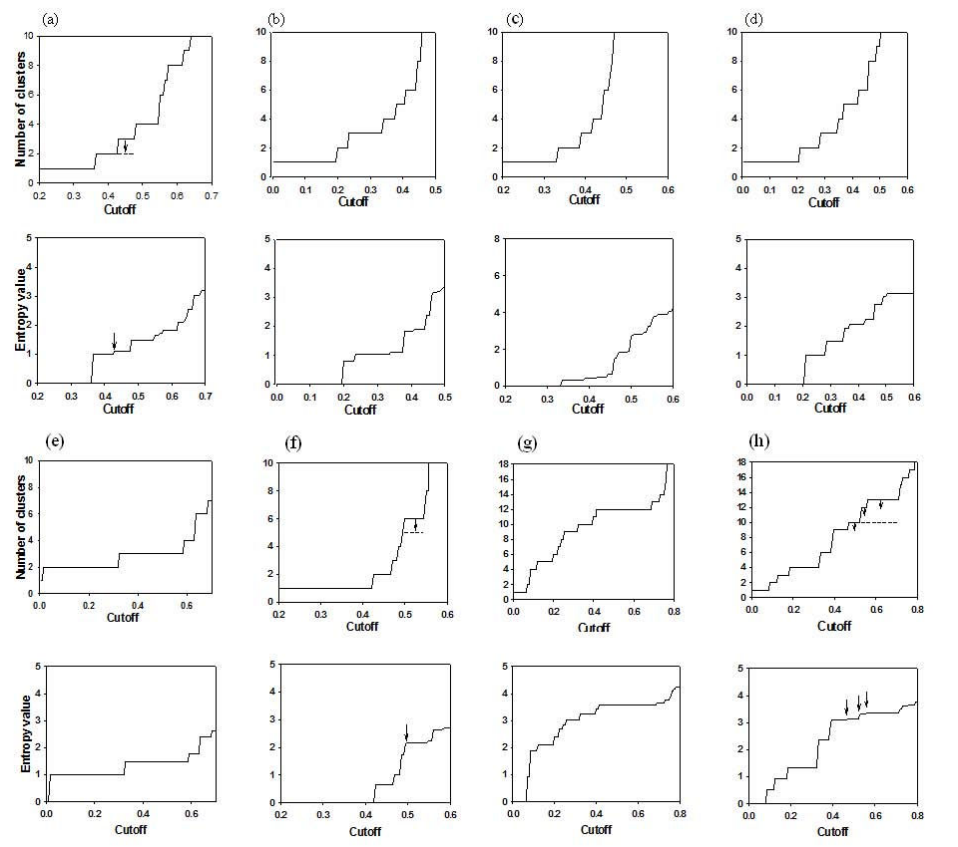
**Cut and entropy-plots for the eight microarray gene expression data sets**: (a) Leukemia, (b) Lymphoma, (c) Colon cancer, (d) Tyroid I, (e) Tyroid II, (f) St. Jude, (g) Normal I, and (h) Normal II. Upper figures represent cut-plots and lower figures, entropy-plots for each data set. Cut-intervals in three cut-plots (a), (f), and (h) were merged as indicated by arrows. The dotted intervals indicate the finalized numbers of clusters. The negligible entropy jumps were also indicated by vertical arrows in entropy-plots.

Figure 2 shows the cut-plots and the entropy-plots for the tested data sets demonstrating how entropy-plots can amend the predictions of cut-plots. Both plots are represented by monotonically increasing step functions and share the jumping points. Each jump in the plots represents separation of a cluster from the former cluster structure (a partition of data points). If a jump in the entropy-plot is smaller than a threshold (0.1/increased number of clusters), we regard the separation of a cluster is negligibly small and suppress the separation. Accordingly, we merge the corresponding adjacent intervals in the cut-plot. The cut-plots shown in Figure 2 are those before such modifications. After modifying the separation of small clusters, the final cluster number and memberships are uniquely determined at the longest cut-plot interval (except *k *= 1). This criterion is commonly applied in comparing the performance of MULTI-K with other algorithms. However, in an explorative study, we recommend investigating the next long cut-plot intervals and the associated cluster structures as well.

The test results are summarized in Table 4. The ARI values in this table represent the agreements between the predicted and the gold standard partitions. MULTI-K overall performed best in both predicting the number of clusters and the accuracy of classification. To our surprise, MULTI-K perfectly classified five of them. The cut-plot of the leukemia data [6] (Fig. 2(a)) had intermediately long flat intervals at two, three, and four clusters. However, at the second jump, the entropy-plot showed a very small increase (0.09), which was caused by the separation of a singleton set. Therefore, we suppressed the partitioning of the singleton set, which merged the second and third intervals. This consequently indicated two major subclasses, which perfectly matched to the two known leukemia classes, ALL(47) and AML(25). By the third jump in the cut-plot, the ALL class was again clearly divided into two known subtypes, ALL-B(38) and ALL-T(9). Although the modified cut-plot indicated two major subclasses, MULTI-K was able to unveil further known subtypes clearly.

**Table 4:** Test results for real expression data sets.

Data set	MULTI-K	GCCk	GCCc	*k*-means		Hier.	
				
				gap	Sil	gap	Sil
Leukemia (**3**)	**0.7364 **(2)	**0.7284 **(4)	**0.7040 **(2)	0.4604 (6)	0.6930 (2)	**0.7521 **(4)	0.4881 (2)
Lymphoma (**3**)	**1.0000 **(**3**)	0.3016 (5)	**0.9414 **(2)	0.1454 (6)	**0.8943 **(2)	**0.9414 **(2)	**0.9414 **(2)
Colon (**2**)	**1.0000 **(**2**)	0.0349 (5)	0.0386 (4)	0.0540 (6)	0.0088 (25)	**0.8944 **(**2**)	**0.8944 **(**2**)
Thyroid I (**2**)	**1.0000 **(**2**)	0.2396 (5)	0.1351 (4)	**0.7335 **(3)	**0.7772 **(**2**)	0.3462 (3)	0.4183 (2)
Thyroid II (**2**)	**1.0000 **(**2**)	0.4468 (4)	0.3986 (5)	**1.0000 **(**2**)	**1.0000 **(**2**)	0.5895 (4)	**1.0000 (2)**
St. Jude (**6**)	**0.8015 **(5)	**0.7490 **(5)	0.6697 (4)	0.5579 (9)	0.1985 (2)	0.1985 (2)	0.1985 (2)
Normal I (**12**)	**1.0000 **(**12**)	**1.0000 **(**12**)	0.4964 (11)	**0.9546 **(13)	**0.7356 **(10)	0 (1)	**1.0000 (12)**
Normal II (**13**)	**0.7977 **(10)	**0.7629 **(11)	0.2476 (13)	**0.7650 **(15)	0.6875 (10)	0.1985 (2)	0.6830 (16)

In each parenthesis is shown the known (left header) and the predicted number of clusters. ARI values that exceed 0.7 are shown in bold.

**Figure 3:**
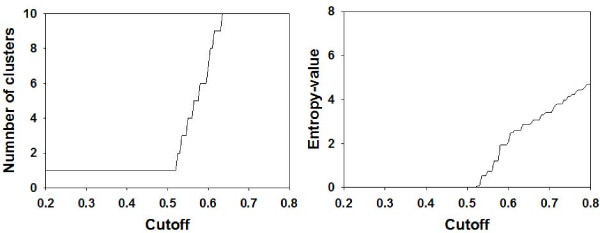
**The cut-plot (left) and entropy-plot (right) for a randomized leukemia data set**.

We then tested MULTI-K on a randomized data set. We randomly permuted each gene's profile of the leukemia data independently. The resulting cut-plot and entropy-plot are shown in Figure 3, where no meaningful intervals or jumps are found. This permutation test demonstrates the existence of cluster structure in the real data.

In analyzing the lymphoma data set [30], the cut-plot had the longest interval at three clusters (except for the one cluster interval) (Fig. 2(b)). The first jump caused a major increase in the entropy value (0.79) and the second jump a minor increase (0.21), both of which were meaningful values (>0.1/increased number of clusters). In the first jump, the 89 samples were clearly divided into two classes, DLBCL(68) and CLL(12)-FL(9) groups. In the second jump, the latter group was again clearly divided into CLL and FL groups. The split of the small subgroup FL caused a relatively small increase in the entropy-plot.

While the entropy-plot modified the predictions of the number of clusters for the leukemia, St. Jude (Fig. 2(f)) [31], and the normal II data sets (Fig. 2(h)) [32], the cut-plot alone correctly identified the number of classes for the other data sets. However, the entropy-plot still provides important information on the impacts of new subdivisions in the clusters. For example, the cut-plot of the thyroid I data [33] had two similarly long intervals at two and three clusters (even though the former is slightly longer) (Fig. 2(d)). Between them, however, the entropy-plot indicated much higher impact of the former subdivision, and hence two major subclasses. Nevertheless, in the second jump, the cancer class was divided into two subsets with six and three elements so that the latter interval might indicate cancer subtypes.

The colon data had a large difference in the sizes of the two classes such that the cancer class had 100 samples while the normal class had only five samples. Even in such a case, MULTI-K clearly separated the two classes. The hierarchical clustering algorithm showed a good performance but the other methods yielded very poor classification rates. The thyroid II data was the most clearly separated so that MULTI-K and *k*-means algorithm as well as the hierarchical-Silhuette methods clearly separated the classes. However, GCC methods failed to indicate the correct numbers and hence yielded low classification rates. On the other hand, for the St. Jude data set, GCC methods performed the second best, while the usual clustering methods performed very poorly. The cut-plot of St. Jude initially indicated six classes but was modified to five after suppressing the separation of a singleton set, though the corresponding jump is too small to be recognized in the entropy graph.

For the last two examples, we chose two data sets with many classes. The normal I data [34] had twelve classes sampled from normal human tissues each of which equally had two samples. The twelve classes in this data set were relatively clearly separated among others so that MULTI-K, GCCk, and hierarchical-Silhuette method clearly identified the known twelve classes, and *k*-means also exhibited good classification rates. The normal II data had thirteen classes, and MULTI-K and GCCk showed similarly good performances.

### Tests for real expression data with fixed known number of classes

As seen in the leukemia example, the 'known' classes also had a hierarchical structure so that it is rather controversial to define 'gold standard sub-classes' because they are merely representing the current level of our knowledge. Moreover, since most algorithms other than MULTI-K failed to indicate the correct number of clusters in many data sets, it is not clear at this point to address the accuracy of the clustering strategies themselves. In other words, the tested algorithms could have yielded better performance combined with other possible indicators for the number of clusters. For these reasons, we investigate the performance of the clustering strategies themselves by specifying the 'known' number of clusters in each algorithm. The test results are shown in Table 5. In this analysis, most clustering strategies improved the ARI values for some data sets, but MULTI-K still was the best method for all the data sets so that it perfectly classified six data sets. As we have analyzed for the leukemia data, MULTI-K perfectly classified the three known sub-classes, while the other methods still misclassified some samples. The thyroid II data set was most clearly classified so that all the algorithms identified the underlying two classes precisely.

**Table 5:** Test results for known number of classes.

Data set	MULTI-K	GCCk	GCCc	*k*-means	Hier.
Lymphoma	**1.0000**	0.4027	**0.9893**	**0.9207**	**0.9058**
Colon	**1.0000**	0.0252	0.0342	0.0390	**0.8944**
Thyroid I	**1.0000**	**0.7772**	0.5815	0.4138	**0.7772**
Thyroid II	**1.0000**	**1.0000**	**1.0000**	**1.0000**	**1.0000**
St. Jude	**0.8570**	**0.7490**	0.6352	**0.7598**	0.1958
Normal I	**1.0000**	**1.0000**	0.4544	**0.8054**	**1.0000**
Normal II	**0.7830**	**0.7210**	0.4590	**0.7364**	0.4934

ARI values that exceed 0.7 are shown in bold

The hierarchical method, if not perfect, has been known to classify the leukemia data quite well [7], but the ARI value was poor (0.4723) in this test. This was caused by the separation of a singleton set, and hence we tried four clusters and assigned the singleton set to the nearest cluster among the other three clusters. This post-processing yielded a much better classification rate of 0.7680 for the leukemia data set. This also illustrates why data processing such as entropy-plot is required. Some other data sets were also better classified by this process so that the lymphoma and normal II data sets had improved ARI values 1.0000 and 0.6653, respectively. However, the modified hierarchical clustering did not outperform MULTI-K in any data set.

### Analysis of breast cancer data without gold-standard known subtypes

Breast cancer has been frequently investigated of its subtypes using gene expression profiles. Different subtypes predicted from hierarchical clustering of expression profiles exhibited different clinical prognoses, which suggests breast cancer is separable into distinct disease. Sorlie et al. [35] compiled 122 breast tissue samples as well as ~500 genes intrinsic to the disease to predict five cancer subtypes. They also extracted five core sample groups that are most highly correlated in each subgroup. We re-analyzed the breast cancer data set from Sorlie et al. [35]. Because the previous subtypes had been inferred over the 'intrinsic' genes, we used the core sample groups in each subtype as the 'silver' standard. Because many kinds of disease are un-informative of such 'intrinsic genes', it is important to reproduce the previous result without functional information of genes. Therefore, we chose 300 high-variance genes in a fully unsupervised manner and compared the performance of the hierarchical clustering and MULTI-K. To compare with the previous prediction, we chose four large entropy jumps to partition the data into five clusters. Four of the previous subtypes largely agreed with the MULTI-K clusters but two of them (Luminal A and ERBB) were merged into one cluster with overall ARI = 0.3704. On the other hand, the hierarchical clustering completely failed to reproduce the previous subtypes and yielded ARI = 0.0803 at maximum (when we chose 25 clusters). In this case, very small clusters were continuously separated from large one cluster as we lower the tree-cut value of the hierarchical clustering. See Additional file 2 for related data.

### Detecting outliers in *MULTI-K*

MULTI-K basically assigns every point to a cluster. However, outliers in each cluster can be identified by computing the average distance of each data point to other points in the cluster. For a cluster *C*_*i *_= {*x*_1_, *x*_2_,..., *x*_*c*_}, let *d*_*j*_, *j *= 1,..., *c*, be the average distance of *x*_*j *_to other points in the cluster, and *mean*_*i *_and *std*_*i *_be their mean and standard deviation. We regard a data point *x*_*j *_as an outlier if |*d*_*j *_- *mean*_*i*_|> *α*·*std*_*i *_where *α *is a positive constant. Using this scheme, we analyzed the clusters for the St. Jude data set that showed a relatively low ARI (0.8015) in our test. When we set *α *= 2 and 1.5, we identified nine and 22 outliers in total, respectively. Excluding those data points, we obtained increased ARI values of 0.8398 and 0.8670, respectively. This implies class assignment by MULTI-K can be improved by removing outliers.

Overall, MULTI-K exhibited consistently good performance, while the performance of the other methods varied much depending on the data set, the clustering algorithm employed, or the indicator function chosen.

## Discussion

Identifying subclasses of diseases using microarray data is a clinically important and computationally challenging problem. The basic assumption of the problem is that distinct subtypes, if any, are separated among others in a high dimensional sample space, and hence can be identified through computational methods: Although the differences in each dimension may be small, they will achieve clear separations if accumulated in a very high dimensional space: The simulation tests for H2 and H3 have been designed in this perspective. Indeed, as shown in the test examples, the ordinary clustering methods successfully identified the known subclasses in some data sets. To improve the performance, ensemble or re-sampling based clustering techniques have been developed [16,18,20,21,36]. Ensemble learning techniques have been widely used in genomic data analysis such as prediction of protein-protein interactions, alpha-membrane proteins [37], protein fold pattern recognition [38], learning the structure of genetic networks from time-series expression data [39] as well as microarray data classification [36,40].

In this article, we presented a cluster-number-based ensemble clustering algorithm, MULTI-K, and suggested using it for unsupervised classification of microarray samples. Unlike other widely used clustering methods, MULTI-K was able to identify clusters with complicated geometric structures as well as high dimensional and noisy clusters. It demonstrated outstanding performance in various simulated and real expression data sets for subtype classification. We note that the Gustafson-Kessel (G-K) clustering algorithm [41] also targets clusters with non-compact shapes, but G-K method mainly focuses on linear cluster structures and tends to cause a numerical problem in computing the eigen-structure of covariance matrices for high-dimensional data. Moreover, G-K method itself does not suggest the optimal number of clusters.

The average linkage hierarchical and *k*-means clustering methods are designed to capture compact or relatively simple clusters. However, the geometric features of the microarray clusters are hardly characterized because they reside in a very high dimensional space and are affected by various sources of noise as well as potential gene interactions. Our tests showed that previous clustering methods that focused on compact clusters yielded poor predictions in many data sets. On the other hand, the suggested method exhibited significant superiority and perfectly classified five (or six) real expression data sets out of eight, while the other methods perfectly classified at most two. We infer the flexibility of MULTI-K in both geometric complexity and high-dimensionality enabled the accurate cclassification of gene expression data.

MULTI-K provides two forms of useful exposition of cluster structure, the cut-plot and the entropy-plot, that inform the hierarchical structure and the natural number of clusters. This pair of indicators is much more informative than other internal indicators most of which suggest only the number of clusters. As shown in the above examples, the cut-plot and entropy-plot give a portrait of the overall cluster organization in a complementary manner, which provides researchers with a rich source of information to decide what the appropriate clusters are. Indeed, use of this pair of indicators outperformed other widely used indicators in various tests.

One possible weak point with MULTI-K is the existence of the free parameter relative to *D*, the distribution on the number of clusters. However, the algorithm showed reliable performances with the rule suggested in this study. To the authors' knowledge, most ensemble learning methods include free parameters, whose basic principle is that the ensemble methodologies improve the performance for a wide range of the free parameters.

An important related topic about sample classification is the gene selection problem. The performance of clustering usually varies more or less depending on the gene subsets chosen. We have commonly used same number of genes with high variance. One possible method in our ensemble context is simultaneously randomizing the number of high variance genes as well as the number of clusters in constructing the weighted graph. However, this approach has not facilitated some meaningful improvements in our experiments (data not shown). Further extensive tests and investigation on gene selection problem is required.

## Conclusion

We found the geometric complexity is most important feature of clusters for accurate classification of microarray samples, which has been often overlooked by other clustering methods. MULTI-K exploits the geometric information of clusters very well since it applies ensemble clustering by varying the number of clusters. With its high performance and simplicity, we expect MULTI-K will become a useful method to uncover the subtypes of disease from expression profiles.

## Methods

Formal statement of the MULTI-K algorithm

Inputs:

1) A set *S *of *N *points in R^n^

2) A number *M *of clustering runs to perform.

3) A distribution *D *of numbers of clusters.

Outputs:

1) A category function *CAT*: *S *→ *Z*.

2) A cut- plot *f*_*cut*_: [*0*, *1*] → *Z*

Details:

   Initialize an *N *× *N *matrix *W *of pair-wise connection strengths to contain all zeros.

   Repeat *M *times

      Select an integer *d *from *D*

      k-means cluster S with *d *clusters.

      For each {*i*, *j *∈ *S *× *S *with *i*, *j *in the same cluster.

         Increment *W *[*i*] [*j*]

      end For

   end Repeat

   Normalize *W *[*i*] [*j*] by dividing each entry of *W *[*i*] [*j*] by *M*

   For *l *equals *0 *to *M*

         Construct graph *G *with *V*(*G*) = *S*, *E*(*G*) pairs of points for which *W *[*i*] [*j*] *>l*/*M*

         Compute number of connected components *c *of *G*

         Add the point (*l*/*M, c*) to *f*_*cut*_

         Compute

            

   end For

   For *x *with *l*/*M <x <*(*l+1*)/*M*, *f*(*x*) = *f*(*l*/*M*) and *H*(*x*) = *H*(*l*/*M*)

      Build a new graph on *S *with edges where *W *[*i*] [*j*] >C

      *C *is chosen such that *f *= *f*(*c*) ≠ 1}{*f *= *f*(*C*) ≠ 1} has the longest length.

   Enumerate the connected components of this graph.

      *CAT *[*i*] is the number of the connected component containing *i*.

### Convergence of the MULTI-K algorithm

MULTI-K is a stochastic algorithm and the cut-plot is its *ad hoc *outcome. We show here the convergence of the algorithm and cut-plot. Suppose that *i*, *j *∈ *S*. Let *p*_*ij *_be the probability that *i *and *j *will be in the same cluster if we pick a number of clusters *d *from the distribution *D *and then cluster *S *using *d *clusters. Since *D *is a distribution on a finite set (more clusters than data items is nonsensical) and the number of possible outcomes of MULTI-K is also finite, there is no problem with the existence of *p*_*ij*_, which is a well-defined probability. Let W* be the graph on vertex set *S *with the edge weight *p*_*ij *_between *i *and *j*. All the zero-weight edges are removed. Let f*(*x*) be the cut-plot derived from W* as a standard cut-plot is derived from *W*. Using the un-normalized version of *W*[*i*][*j*], we obtain lim_*M *→ ∞ _. Therefore, as we increase *M*, MULTI-K creates graph *W *that are successively better approximations to W*. Likewise, cut-plot *f*(*x*) approximates to f*(*x*).

## Authors' contributions

EYK co-developed the algorithm and codes and analyzed data. SYK co-designed the study and aided data analysis. DA co-developed the algorithm and wrote the Additional file 1. DN co-designed and supervised the study, analyzed data, and drafted the manuscript. All the authors contributed to the writing of the main manuscript.

## Supplementary Material

Additional File 1 **Extended explanation of the entropy-plot.** Click here for file

Additional File 2 **Predicted partitions in breast cancer data sets**. Cut & entropy plot for the breast cancer data as well as the partitions used in the main text are shown. Click here for file
