# Supplementary material for: MULTI-K: accurate classification of microarray subtypes using ensemble k-means clustering
Source: BMC Bioinformatics. 2009 Aug 22;10:260. doi: 10.1186/1471-2105-10-260 (PMC2743671; doi:10.1186/1471-2105-10-260)
Supplement: Additional File 1 — Extended explanation of the entropy-plot. [file 1471-2105-10-260-S1.doc]

**Supplementary Material**

**Extended explanation of the entropy-plot**

An issue that arose in the first explorations of multi-clustering is that the cut-plot gives less-than-detailed information about the size of clusters that are forming as the cut-value is increased. The entropy-plot was developed to address this limitation. In brief, the entropy-plot replaces integer-valued jumps that appear each time a cluster is divided with a real-valued jump that reflects the information present in the cluster division. The entropy-plot is based on the information theoretic entropy of the distribution of cluster sizes. Entropy is a standard method of measuring the information present in a probability distribution. In order to compute the entropy of a set of cluster sizes the sizes are normalized by dividing by the total number of points. This gives a probability distribution that measures the empirical probability
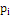
 of a point belonging to a given cluster. The entropy used in the entropy-plot is the entropy of this distribution, given by Equation (1).


Entropy is a measure of information content and so the entropy-plot displays how the information in the cluster structure changes as new clusters form. A simple example that illustrates the value of entropy comes from recording many experiments in which a coin is flipped. In this case the coin has probability p of heads and probability 1 - p of flipping tails. In this situation Equation (1) to simplify to Equation (2), graphed in Supplementary Figure 1. Notice that the maximum information content for a coin is one bit and that this occurs for a fair coin, one with a 50/50 chance of producing a head or tail. This type of coin corresponds, in an information-theoretic sense, to dividing a cluster in half.

Supplementary Figure 1: Entropy of a coin as a function of the coin's bias


Entropy measures the information content of a coin with probability p of heads in the following sense. A fair coin, flipped many times, will generate a random string without bias or pattern. One bit of information is needed to report a flip of this fair coin and compression of the pattern of results produced by this coin is negligible. A coin that has a high probability of heads will generate a string that is mostly heads. This string has low information content and so is easy to compress. In an information-theoretic sense the flips of the biased coin contain far less information than those of the fair coin. Likewise, even division of a large cluster is a more informative event than uneven division. It is worth making the notion of information content precise. The entropy of the coin, given in Equation (2), measures the number of bits required to store the outcomes of flipping the coin (so long as we are storing many flips of the coin). If a coin with probability p of heads is flipped many times, Equation (2) gives a close estimate of how much a long string of flips can be compressed.

We return now to the entropy-plot. If, as we change the cut-value, a cluster divides in half, this creates the maximum number of new relationships between data points and hence the greatest possible increase in information present in the cluster structure of the data. If, on the other hand, a single point splits off of a cluster this represents the smallest possible change in the information present in the cluster structure. Using the entropy-plot permits the user to see the relative importance of different cluster divisions which looked the same in the original cut-plot. As a convenience to the user, we make the process of using the entropy-plot automatic in one simplest way by declining to report tiny clusters. Such clusters are often artefactual and unimportant, cluttering the output with insignificant information.
